# Cognitive deficits in childhood, adolescence and adulthood in 22q11.2 deletion syndrome and association with psychopathology

**DOI:** 10.1038/s41398-020-0736-7

**Published:** 2020-02-03

**Authors:** Sinead Morrison, Samuel J. R. A. Chawner, Therese A. M. J. van Amelsvoort, Ann Swillen, Claudia Vingerhoets, Elfi Vergaelen, David E. J. Linden, Stefanie Linden, Michael J. Owen, Marianne B. M. van den Bree

**Affiliations:** 1 Medical Research Council Centre for Neuropsychiatric Genetics and Genomics, Division of Psychological Medicine and Clinical Neurosciences, Cardiff University, Cardiff, Wales UK; 2 Department of Psychiatry and Psychology, Maastricht University, Maastricht, The Netherlands; 3 Department of Human Genetics, Faculty of Medicine, KU Leuven, Leuven, Belgium; 4 Center for Human Genetics, University Hospitals Leuven, Leuven, Belgium; 5 Department of Radiology and Nuclear Medicine, Amsterdam University Medical Centers (Location Academic Medical Center), Amsterdam, The Netherlands; 6 University Psychiatric Center KU Leuven, University Hospital Leuven, Leuven, Belgium

**Keywords:** Human behaviour, Medical genetics

## Abstract

22q11.2 Deletion Syndrome (22q11.2DS) is associated with high risk of psychiatric disorders and cognitive impairment. It remains unclear to what extent key cognitive skills are associated with psychopathology, and whether cognition is stable over time in 22q11.2DS. 236 children, adolescents and adults with 22q11.2DS and 106 typically developing controls were recruited from three sites across Europe. Measures of IQ, processing speed, sustained attention, spatial working memory and psychiatric assessments were completed. Cognitive performance in individuals was calculated relative to controls in different age groups (children (6–9 years), adolescents (10–17 years), adults (18+ years)). Individuals with 22q11.2DS exhibited cognitive impairment and higher rates of psychiatric disorders compared to typically developing controls. Presence of Autism Spectrum Disorder symptoms was associated with greater deficits in processing speed, sustained attention and working memory in adolescents but not children. Attention deficit hyperactivity disorder in children and adolescents and psychotic disorder in adulthood was associated with sustained attention impairment. Processing speed and working memory were more impaired in children and adults with 22q11.2DS respectively, whereas the deficit in sustained attention was present from childhood and remained static over developmental stages. Psychopathology was associated with cognitive profile of individuals with 22q11.2DS in an age-specific and domain-specific manner. Furthermore, magnitude of cognitive impairment differed by developmental stage in 22q11.2DS and the pattern differed by domain.

## Introduction

22q11.2 Deletion syndrome (22q11.2DS) is associated with a range of psychiatric disorders, with over half of individuals reaching criteria for at least one psychiatric diagnosis across the lifespan^1,2^. Most strikingly, in adult samples, ~40% are diagnosed with a psychotic disorder including schizophrenia^3,4^, although it must be noted that this may reflect ascertainment bias as more recent population estimates are much lower^5^. Several studies have established a high prevalence of childhood psychopathology manifesting as attention deficit hyperactivity disorder (ADHD), autism spectrum disorder (ASD) and anxiety disorders^2,3^. Prevalence of psychiatric disorders appears to follow different trajectories over the lifespan, such as the rate of anxiety disorders remaining relatively stable across developmental stages^6^, whereas the rate of ADHD diagnosis declines, with 33–43% individuals no longer reaching diagnostic criteria at follow-up in adolescence^7,8^. Therefore, it is important to take a developmental perspective when considering psychiatric disorders in 22q11.2DS, which reflects the dynamic development of the brain.

22q11.2DS is highly penetrant for a broad range of cognitive impairments^6,9,10^. It is important to investigate whether the cognitive deficits in 22q11.2DS are stable over development, or if they are worse at certain developmental time points, as looking at cognition across the life span in a high-risk group for psychopathology can provide important insights into underlying mechanisms^11^.

Studies investigating associations between cognition and psychopathology in young people with 22q11.2DS have produced mixed results. Some previous research has suggested that cognitive deficits and psychopathology constitute distinct pleiotropic outcomes of the deletion^2,12^. Other research has suggested that cognition may mediate psychopathology, for example, that deficits in executive functioning may index vulnerability to ASD or ADHD in children with 22q11.2DS^13^. Conversely, reverse causation has been proposed, with the findings that presence of anxiety symptoms may mediate the relationship between 22q11.2DS and working memory capacity, with greater anxiety linked to less efficient cognitive performance^14^. Other research has reported that the presence of ASD or ADHD may predict poorer executive functioning in 22q11.2DS^15^. It has furthermore been suggested that age may play a role in these complex relationships between cognitive deficits and psychopathology^13^. To date, we are not aware of any papers in 22q11.2DS reporting on associations in cognition and psychopathology across developmental stages (i.e., children and adolescents), which has been proposed as an important topic for future research^13^.

Research on the relationship between cognition and psychopathology in adults with 22q11.2DS has largely focused on associations with psychotic disorders. Individuals with 22q11.2DS and schizophrenia have been reported to display greater deficits on measures of executive function such as working memory and sustained attention than individuals with 22q11.2DS without schizophrenia^16–18^, conforming to studies of schizophrenia in individuals without a genetic syndrome^19^. Additionally, longitudinal studies following adolescents with 22q11.2DS into adulthood found that IQ (particularly verbal IQ) was lower in those with psychotic disorders than those without psychotic disorder^6,20^. A cross-sectional study of adults found deficits in verbal learning, social cognition and set-shifting between adults with 22q11.2DS and schizophrenia compared to those without schizophrenia, but did not find this difference on attentional measures, suggesting that attentional dysfunction may be a general feature of 22q11.2DS over and above the influence of psychosis^21^. Despite this, inattention symptoms have been proposed to have an important role in psychosis in 22q11.2DS^22^. Therefore, there is still debate as to which cognitive functions are associated with psychotic disorders in 22q11.2DS.

The literature on cognitive trajectories from childhood to adulthood in 22q11.2DS is also inconsistent. Some studies have reported evidence of cognitive decline as individuals get older, with studies focussing on IQ reporting negative associations with age both cross-sectionally^12^ and longitudinally^23^. Other longitudinal studies comparing children with the deletion with a comparison sample of typically developing siblings assessed with the same measures have not found evidence of cognitive decline for IQ or a range of neurocognitive functions that have been associated with psychiatric disorder, including processing speed, sustained attention and working memory^9,24^, although there may be developmental lags in specific domains (i.e., children with 22q11.2DS lag behind controls without the deletion). Furthermore, it has been reported that longitudinal cognitive profiles were similar between children with 22q11.2DS and IQ-matched and age-matched children with idiopathic intellectual disabilities (IID; Van Den Heuvel^25^). Examining impairments in specific cognitive domains such as attention may provide insights into neurobiological processes^11^ and that these functions may be more sensitive both to cognitive change and to remediation strategies than a global measure of intelligence like IQ^26^.

The majority of the studies of specific cognitive domains in 22q11.2DS have focussed on children and adolescents although it is recognised that many cognitive functions continue to mature through early adulthood^27,28^. Our understanding of cognitive function in adults with the deletion thus remains limited^29–31^. One study found that adults with 22q11.2DS exhibited deficits in visuoperceptual, planning, abstract, and social cognition compared to age-matched, gender-matched, and IQ-matched controls, which further reinforces the importance of considering specific cognitive domains beyond a sole focus on IQ^32^. When examining change over a wider age range up to 26 years, one study found that overall working memory trajectory in 22q11.2DS differed from typically developing controls whereas other domains such as inhibition followed the same developmental course^28^. It was therefore proposed that individuals with 22q11.2DS may reach a “developmental plateau” in working memory ability before controls, but this was only observable because of the inclusion of older adolescents and adults^28^. However, another study with a similar age range investigating working memory abilities found that individuals with 22q11.2DS caught up with controls by age 25^26^. A recent meta-analysis of cognitive functioning in 22q11.2DS compared to controls reported that in paediatric samples there was evidence for greater cognitive impairment in older children, but in adulthood there was an improvement in cognitive abilities over time^31^, highlighting the importance of looking over a wide age range to understand better the continuing development of cognitive functions in 22q11.2DS. Furthermore, it is a general methodological limitation of previous research that different studies with relatively small numbers of participants have used different cognitive testing batteries, limiting comparisons that can be made across samples^31^. There is a need for testing a range of cognitive domains in a large sample where all participants have completed the same battery^17^.

This study took a developmental approach with a consistent cognitive battery across international sites with the primary aim to examine associations between cognitive performance and psychopathology. Specifically, in young individuals with 22q11.2DS we investigated whether any links with cognitive performance and the presence of ASD, ADHD or anxiety disorders differed for children and adolescents. In adults, we examined the association of cognition with the presence of a psychotic disorder. Based on the majority of previous research, we hypothesised that presence of a psychiatric disorder would be associated with greater cognitive deficits. The second aim was to assess whether cognitive functioning differed across the three developmental stages in 22q11.2DS (childhood, adolescence, and adulthood) compared to typically developing individuals. Previous research suggests that we may expect greater cognitive impairments in older individuals compared to younger individuals, at least in some domains.

## Methods

### Participants

Three hundred and forty-two participants (236 individuals with 22q11.2DS and 106 controls) were recruited from three sites across Europe (see Supplementary Table 1). Cardiff University recruited children and adolescents with 22q11.2DS and their typically developing control siblings, and adults with 22q11.2DS through the Experiences of CHildren with cOpy number variants (ECHO) and Defining Endophenotypes From Integrated Neuroscience (DEFINE) studies. KU Leuven recruited adolescents and adults with 22q11.2DS and Maastricht University recruited adults with 22q11.2DS and adult community controls. Participants were ascertained through similar recruitment methods, which was not on the basis of psychiatric presentation but largely through medical genetics services after receiving a diagnosis of 22q11.2DS (see Supplementary Table 1 for further details). Children were defined as 6–9.9 years old, adolescents as 10–17.9 years old and adults 18+ following World Health Organisation guidelines^33^. Mean ages were comparable in 22q11.2DS and control groups within the adolescent and adult developmental stages; however, in the child stage the 22q11.2DS group were on average a few months younger (see Supplementary Table 2). Gender distributions were comparable in the child and adolescent developmental stages but there were significantly more female participants in the adult stage (see Supplementary Table 2).

Fifty-four participants (1 child, 3 adolescents and 50 adults) were taking neuroactive and/or thyroid medication at the time of testing (see Supplementary Table 3 for further details). The authors assert that all procedures contributing to this work comply with the ethical standards of the relevant national and institutional committees on human experimentation and with the Helsinki Declaration of 1975, as revised in 2008. Written informed consent was gained from primary carers and participants. The study was approved by ethics committees at the three sites (Cardiff, NHS Wales Research Ethics Committee; Maastricht, Maastricht University Medical Centre; KU Leuven; Research Medical Ethics Committee UZ KU Leuven).

### Cognitive assessments

A battery of three neurocognitive tasks comparable across sites was administered using Cambridge Neuropsychological Test Automated Battery (CANTAB) software^34^. Given the high risk of neurodevelopmental disorders in 22q11.2DS, cognitive tests were chosen that would be sensitive to deficits associated with these disorders (ADHD^35^, autism^36^, schizophrenia^37^). These measured processing speed, sustained attention and spatial working memory (see Supplementary Table 4 for full task details). CANTAB raw scores were transformed into age-standardised *z*-scores based on normative data. Full scale IQ (FSIQ), verbal IQ (VIQ), and performance IQ (PIQ) were assessed at each site with Wechsler scales in validated local language versions (see Supplementary Table 5 for full details). IQ scores were calculated by standardising raw scores for age based on normative data. Not all participants completed all cognitive tasks, due to cognitive or behavioural issues or time constraints, but all completed at least one; this is detailed in Table 1.

**Table 1:** Differences in cognitive performance between individuals with 22q11.2DS and typically developing controls for the three developmental stages.

	Child						Adolescent							Adult							
	*22q11.2DS*		Control					*22q11.2DS*		Control					*22q11.2DS*		Control				
	*n*	Mean (sd)	*n*	Mean (sd)	*F* (df)	*p*	Effect size; *η*_p_^2^	*n*	Mean (sd)	*n*	Mean (sd)	*F* (df)	*p*	Effect size; *η*_p_^2^	*n*	Mean (sd)	*n*	Mean (sd)	*t* (df)	*p*	Effect size; Cohen’s *d*
Neurocognitive *z*-scores																					
Processing speed	55	−0.64 (2.19)	22	0.58 (0.77)	11.74 (1,11)	**0.006**	0.08	60	0.02 (1.66)	34	0.55 (0.75)	4.3 (1,14)	0.057	0.03	99	0.49 (1.44)	48	1.02 (0.45)	3.32 (130.48)	**0.001**	0.43
Sustained attention	49	−2.93 (2.36)	20	−0.68 (1.20)	57.5 (1,10)	**<0.001**	0.20	56	−1.77 (4.24)	34	−0.08 (1.22)	53.62 (1,14)	**<0.001**	0.05	86	−1.70 (1.12)	48	0.52 (0.81)	13.17 (123.79)	**<0.001**	2.16
Working memory	60	−0.89 (1.02)	23	0.21 (0.88)	40.22 (1,12)	**<0.001**	0.20	65	−1.21 (0.84)	36	−0.50 (1.03)	19.51 (1,16)	**<0.001**	0.13	106	−0.41 (0.89)	48	1.20 (0.32)	16.45 (146.53)	**<0.001**	2.12
IQ test scores																					
Full-scale IQ	58	79.71 (10.05)	22	114.50 (16.29)	135.38 (1,11)	**<0.001**	0.63	65	72.90 (13.35)	35	104.49 (11.45)	326.11 (1,15)	**<0.001**	0.59	104	73.47 (11.98)	48	111.42 (16.87)	14.04 (69.72)	**<0.001**	2.77
Verbal IQ	58	81.40 (12.33)	22	116.05 (14.00)	195.88 (1,11)	**<0.001**	0.6	65	74.71 (13.80)	35	104.09 (10.26)	281.31 (1,15)	**<0.001**	0.56	102	72.97 (14.53)	48	108.10 (16.67)	13.17 (148)	**<0.001**	2.3
Performance IQ	60	81.52 (9.29)	22	109.73 (17.52)	70.79 (1,11)	**<0.001**	0.53	65	75.12 (13.06)	35	104.17 (14.17)	256.53 (1,15)	**<0.001**	0.52	102	72.26 (15.68)	48	110.08 (21.65)	10.84 (71.04)	**<0.001**	2.13

*p* values that are statistically significant are shown in bold.

### Psychiatric assessments

Details of the psychiatric assessment of children and adolescents have been previously reported^2^; for further details see Supplementary Table 5. Assessment details for adult participants are also reported in Supplementary Table 5. Instruments were comparable across sites and provided DSM-IV diagnoses of psychiatric disorders including schizophrenia, schizoaffective disorder and psychotic disorder. Diagnoses at each of the three sites were made during consensus meetings led by a psychiatrist. All three centres that have contributed data to this paper are part of the International 22q11.2 Deletion Syndrome Brain Behaviour Consortium (IBBC), which used careful evaluation procedures to ensure phenotypic harmonisation across sites (described in detail in the ref. ^38^).

### Analysis

Mean neurocognitive *z*-scores and IQ scores were compared between individuals with 22q11.2DS and the control sample in the separate developmental stages. Children and adolescents were compared against sibling controls with ANOVA with covariate for relatedness, and adults were compared against community controls with *t*-tests (with correction for unequal variance if applicable).

Mean FSIQ, VIQ, and PIQ scores were compared between sibling and community controls with *t*-tests (with correction for unequal variance if applicable) to investigate whether these different groups of typically developing individuals performed differently.

Within each developmental stage the mean score for the control sample (typically developing siblings for the children and adolescents and community controls for the adults) was subtracted from the score of each individual with 22q11.2DS for each cognitive measure. This produced a difference score for each individual with 22q11.2DS on each measure. A difference of 0 would therefore represent no difference between the individual with 22q11.2DS and the control mean and a negative difference would represent an impairment on that measure in the individual with 22q11.2DS compared to the mean control performance. This enabled the analysis to take into account control performance while comparing performance across age groups (i.e., it enabled us to focus on variation in cognitive performance that can be attributed to the 22q11.2 deletion).

### Aim 1: associations of cognition and psychopathology in the three developmental stages in 22q11.2DS

No children or adolescents met criteria for psychotic disorder at the time of assessment. One adolescent had been diagnosed with a psychotic disorder in the past which was currently managed with antipsychotic medication. Two other adolescents and one child were taking medication, which could have affected cognitive performance (see Supplementary Table 3), so sensitivity analyses were run for each analysis these four participants were included in. 2/60 children and 8/64 adolescents with 22q11.2DS reported subthreshold psychotic phenomena. As these numbers were small, comparisons in cognition between those with and without psychotic disorder/experiences would not have been reliable. However, we included psychotic experiences as a covariate in analyses to establish whether this altered results.

Chi-square tests were conducted to establish whether prevalence of probable ASD, ADHD or anxiety disorder differed in children and adolescents with 22q11.2DS compared to their typically developing siblings.

To investigate whether childhood psychopathology was associated with greater cognitive impairment in childhood or adolescence 2 × 2 ANOVAS were conducted to test for interaction effects (i.e., child vs. adolescent × diagnosis present or absent) and main effects of psychopathology (i.e., diagnosis present or absent).

To compare performance on cognitive measures between adults with psychotic disorder and those without, independent *t*-tests were performed either with or without a correction for unequal variance, as applicable.

### Aim 2: comparing cognitive performance across the three developmental stages in 22q11.2DS

To compare performance on cognitive measures across the three developmental stages, one-way ANOVAs were performed on the difference scores for each cognitive measure. If a significant overall group difference (*p*-value < 0.05) was found, Tukey HSD post hoc tests were conducted to establish which developmental stages differed from each other.

Data analysis was conducted using R version 3.5.0. For code availability please contact the first author. All analyses were also conducted with site as a covariate to control for differences across sites, such as assessment measures.

### Statistical correction

The Benjamini-Hochberg method for controlling False Discovery Rate (FDR) was used to correct for multiple comparisons^39^. This method ranks *p*-values in a cluster of tests, then divides the rank by the number of tests in the cluster, then multiplying by an acceptable FDR (in this case, 10%, following Crawford^40^) to produce a Benjamini-Hochberg critical value. If the original *p*-value is below the Benjamini-Hochberg critical value, then it survives FDR correction.

## Results

Individuals with 22q11.2DS in every developmental stage showed impairment on all neurocognitive and IQ scores compared to controls except for adolescents in processing speed (Table 1). All comparisons survived FDR correction. There was no difference between sibling and community controls in FSIQ (*p* = 0.322), VIQ (*p* = 0.841) or PIQ (*p* = 0.318).

### Aim 1: associations of cognition and psychopathology in the three developmental stages in 22q11.2DS

#### Children and adolescents

Children with 22q11.2DS had higher rates of probable ASD, ADHD, and anxiety disorders compared to their typically developing siblings (Supplementary Table 6). Adolescents with 22q11.2DS had higher rates of probable ASD and ADHD compared to their typically developing siblings (Supplementary Table 6). Cognitive difference scores for children and adolescents with 22q11.2DS with or without probable ASD, ADHD, or anxiety disorder are displayed in Supplementary Tables 7–9, respectively.

#### Probable ASD

Interaction effects between probable ASD status and developmental stage were present on all neurocognitive measures (Table 2). Adolescents with probable ASD appeared to display a greater deficit in these domains compared to adolescents without ASD, but this did not appear to be the case for children (Supplementary Table 7; Supplementary Fig. 1). There were no interaction effects between probable ASD status and developmental stage on IQ measures (Table 2). There were no main effects of probable ASD on IQ measures. Most comparisons survived FDR correction (Table 2).

**Table 2:** Association of cognition and psychopathology in children and adolescents with 22q11.2DS relative to typically developing controls.

	Probable ASD						ADHD						*Anxiety Disorder*					
	Main effect on measure			Interaction with developmental stage			Main effect on measure			Interaction with developmental stage			Main effect on measure			Interaction with developmental stage		
	*F* (df)	*p*	Effect size; *η*²	*F* (df)	*p*	Effect size; *η*²	*F* (df)	*p*	Effect size; *η*²	*F* (df)	*p*	Effect size; *η*²	*F* (df)	*p*	Effect size; *η*²	*F* (df)	*p*	Effect size; *η*²
Neuro-cognitive scores																		
Processing speed	0.59 (1,107)	0.443	0.01	5.45 (1,107)	**0.021**^a^	0.05	2.13 (1,107)	0.147	0.019	1.89 (1,107)	0.172	0.02	0.01 (1,109)	0.916	0	0.44 (1,109)	0.507	0
Sustained attention	2.11 (1,98)	0.149	0.02	5.42 (1,98)	**0.022**	0.05	8.85 (1,98)	**0.004**	0.082	1.61 (1,98)	0.207	0.02	0.02 (1,100)	0.891	0	0.03 (1,100)	0.86	0
Working memory	0.18 (1,116)	0.669	0	8 (1,116)	**0.006**	0.06	2.39 (1,117)	0.125	0.019	0.03 (1,117)	0.865	0	2.29 (1,119)	0.133	0.02	0.76 (1,119)	0.385	0.01
IQ test scores																		
Full-scale IQ	0.53 (1,114)	0.468	0.01	0.54 (1,114)	0.463	0.01	0 (1,115)	0.954	0	2.75 (1,115)	0.1	0.02	0.55 (1,117)	0.46	0.01	0.01 (1,117)	0.905	0
Verbal IQ	1.82 (1,114)	0.18	0.02	0.13 (1,114)	0.723	0	0.08 (1,115)	0.78	0.001	2.46 (1,115)	0.12	0.02	1.46 (1,117)	0.229	0.01	0 (1,117)	0.971	0
Performance IQ	0 (1,116)	0.957	0	1.3 (1,116)	0.257	0.01	0.28 (1,117)	0.598	0.002	2.17 (1,117)	0.143	0.02	0.02 (1,119)	0.891	0	0.19 (1,119)	0.665	0

*ASD* autism spectrum disorder, *ADHD* attention deficit hyperactivity disorder.

^a^Denotes comparison that did not survive multiple comparison correction.

*p* values that are statistically significant are shown in bold.

#### ADHD

There were no interaction effects between ADHD status and developmental stage on neurocognitive or IQ measures (Table 2). There was a main effect of ADHD on sustained attention such that those with ADHD displayed greater impairment regardless of developmental stage (Supplementary Table 8; Supplementary Fig. 2). This survived FDR correction. There were no associations between ADHD and processing speed, working memory or IQ measures.

#### Anxiety disorder

There were no interaction effects between anxiety disorder status and developmental stage or main effects of anxiety disorder status on neurocognitive or IQ measures (Table 2 and Supplementary Table 9).

Sensitivity analyses excluding four participants taking medication which could have affected cognitive performance did not affect results. Furthermore, analyses including presence of subthreshold psychotic experiences as a covariate did not alter results. Additionally, as most of the child and adolescent participants were recruited from the Cardiff site, sensitivity analyses were conducted removing the 5 Leuven adolescents and results were unchanged, suggesting site did not affect performance.

#### Adults

Sixteen percent of the adult sample (*n* = 18) were categorised as having a psychotic disorder; 14 of these had a schizophrenia diagnosis, three a diagnosis of psychotic disorder not otherwise specified (NOS), and one a diagnosis of schizoaffective disorder. None of the control group were diagnosed with a psychotic disorder.

Seven percent of adults without psychotic disorder (*n* = 6) met criteria for prodromal psychotic symptoms. As this was a small number of adults, it was not appropriate to categorise these as a separate group, and so they were classified as adults without psychotic disorder. A sensitivity analysis including presence of prodromal symptoms as a covariate did not change results.

Cognitive difference scores for adults with 22q11.2DS with or without psychotic disorder are displayed in Supplementary Table 10.

Individuals with a psychotic disorder displayed greater impairment in sustained attention and all IQ measures (Table 3 and Supplementary Figs. 3, 4). Conversely, presence of a psychotic disorder was not associated with processing speed or working memory. All comparisons survived FDR correction.

**Table 3:** Association of cognition and psychotic disorder in adults with 22q11.2DS relative to typically developing controls.

	Psychotic disorder		
	*t* (df)	*p*	Effect size; Cohen’s *d*
Neurocognitive scores			
Processing speed	0.61 (97)	0.54	0.17
Sustained attention	3.13 (12.67)	**0.008**	1.29
Working memory	0.26 (104)	0.797	0.07
IQ test scores			
Full-scale IQ	2.43 (102)	**0.017**	0.66
Verbal IQ	2.64 (100)	**0.010**	0.74
Performance IQ	2.03 (100)	**0.045**	0.57

*p* values that are statistically significant are shown in bold.

The association between IQ and sustained attention in adults with 22q11.2DS and psychotic disorder was non-significant (Pearson correlation *r* = 0.32, *p* = 0.344), suggesting that these domains have distinct associations with psychosis.

Fifty adult participants were taking medication which could have affected cognitive performance. As this was a large proportion of the sample, a sensitivity analyses would not have been appropriate, so analyses were repeated controlling for medication as a covariate. Results were unchanged. Additionally, as the adult participants were recruited from three different sites, analyses were conducted with site included as a covariate and results were unchanged.

### Aim 2: comparing cognitive performance across developmental stages in 22q11.2DS

Cognitive difference scores across developmental stages are displayed in Supplementary Table 11. Working memory performance differed across the developmental stages (Table 4). Post hoc tests revealed that adults with 22q11.2DS showed more impairment on this task than children and adolescents. Processing speed differed according to developmental stage with children displaying greater deficits than adults. Impairment in sustained attention appeared to be similar across developmental stages (Supplementary Fig. 5).

**Table 4:** Comparison of associations between developmental stage and cognitive impairment between the three developmental groups.

	ANOVA			Tukey HSD post-hoc tests		
	*F* (df)	*p*	Effect size; *η*_p_^2^	Child and adolescent	Child and adult	Adolescent and adult
Neurocognitive scores						
Processing speed	3.32 (2,211)	**0.038**	0.031	0.080	**0.045**	0.999
Sustained attention	0.82 (2,188)	0.443	0.009	–	–	–
Working memory	20.39 (2,228)	<**0.001**	0.152	0.051^a^	**0.002**	<**0.001**
IQ test scores						
Full-scale IQ	5.74 (2,224)	**0.004**	0.049	0.20	0.644	**0.003**
Verbal IQ	3.81 (2,222)	**0.024**	0.033	0.089	0.975	**0.025**
Performance IQ	13.02 (2,224)	<**0.001**	0.104	0.936	<**0.001**	<**0.001**

^a^When controlling for site as a covariate, this difference was significant (*p* = 0.028).

*p* values that are statistically significant are shown in bold.

There were differences across developmental stages on all measures of IQ (Table 4). Post hoc tests revealed that adults showed greater impairment than adolescents on all measures, and that adults also performed worse than children on PIQ (Supplementary Fig. 6). All comparisons survived FDR correction.

Fifty-four participants were taking medication which could have affected cognitive performance, so analyses were repeated controlling for medication as a covariate. Results were unchanged. When controlling for site as a covariate, results were largely unchanged except there was a significant difference between children and adolescents on working memory which was previously not significant.

All analyses were also ran controlling for gender as a covariate, as it has been previously reported that there may be gender differences in cognitive performance^15^ but this did not change any associations.

## Discussion

To our knowledge, this is the largest study in 22q11.2DS where all participants have completed the same testing battery in the specific cognitive domains of processing speed, sustained attention and working memory, in addition to standard IQ assessments. Furthermore, typically developing individuals (siblings and community controls) were included to account for natural variation in cognition over development. Presence of psychopathology had developmental and domain specific associations with the cognitive profile. The presence of probable ASD, ADHD, and psychotic disorder at specific developmental stages were differentially associated with deficits on specific cognitive domains, suggesting there may be shared brain pathways or potential critical periods for cognitive development.

Cognitive impairments were present across childhood, adolescence, and adulthood in 22q11.2DS compared to typically developing controls, but magnitude of impairment differed by developmental stage and the pattern differed by domain. Processing speed and working memory were more impaired in children and adults with 22q11.2DS respectively, whereas the deficit in sustained attention was present at the same magnitude across all developmental stages. Impairments in working memory and IQ were more salient in adults with 22q11.2DS than children and adolescents.

Rates of probable ASD, ADHD, and anxiety disorder in the current study were comparable to a large previous study of individuals with 22q11.2DS^3^. Presence of probable ASD was found to interact with developmental stage such that adolescents with probable ASD experienced greater impairment than children with probable ASD in processing speed, sustained attention and working memory. Previous research including a subgroup of participants from the current study did not find a relationship between probable ASD and cognitive function^2^ (see Supplementary Table 1). This previous study, however, considered children and adolescents as one group, whereas in the current study the increased sample size enabled comparison between the developmental stages of childhood and adolescence, allowing a developmental perspective on the relationship between ASD and cognition.

These results could imply that cognitive impairment associated with ASD emerges in adolescence and is not present in early childhood. This indicates that there may be a sensitive period for acquiring certain cognitive domains in ASD, whereby individuals with ASD acquire skills well in childhood but this does not continue into adolescence, a theory which is in line with longitudinal studies of young people with autism^41^. It is unclear from this cross-sectional study design whether our findings indicate a developmental lag, where the gap between children and adolescents with ASD increases with age compared to those without ASD, or whether adolescents with ASD are performing worse at a later time point than at baseline, which would index cognitive deterioration (see Chawner^24^). Longitudinal research is vital to make this distinction.

It could also be possible that increasing perceived social and academic demands on individuals with ASD during adolescence result in greater difficulty reaching equivalent mental age to controls than in childhood^41^. Alternatively, it has been proposed that measuring ASD symptoms in young people with 22q11.2DS may be indexing pre-psychotic traits, especially social deficits that are measured by the SCQ^42^. As the presence of such prodromal symptoms has been associated with poorer cognitive functioning in 22q11.2DS^26,28^, an alternative explanation for the association in this study of probable ASD with cognitive deficits in adolescence could be that those categorised with probable ASD are actually manifesting prodromal psychotic symptoms. However, including the presence of subthreshold psychotic experiences as a covariate did not change results. Furthermore, a longitudinal study did not find an association between autistic features in individuals with 22q11.2DS in childhood and their risk of developing psychotic disorders or symptoms^43^ which suggests that both psychiatric conditions may be pleiotropic phenotypes of 22q11.2DS.

However, as the presence of probable ASD was determined by the SCQ which is a screening questionnaire and does not provide a formal research diagnosis, caution must be taken in interpreting these findings. This is a questionnaire which was developed to align to the gold-standard Autism Diagnostic Interview-Revised (ADI-R; Lord, Rutter and Le Couteur^44^) and the two measures correlate highly (Berument, Rutter, Lord, Pickles and Bailey^45^; Charman^46^). However, it is thought that it does not perform as well as the ADI-R when discriminating ASD from intellectual disability (ID; Berument, Rutter, Lord, Pickles and Bailey^45^) so it is possible that the SCQ may be indexing more than ASD only. However, there was no association of IQ with meeting the cut-off for ASD on the SCQ, which suggests the SCQ was not just indexing ID. Furthermore, this criteria has been applied in previous research studies^2,47^. Above all, the finding that children who meet clinical cut-off for social communicative deficits may have different cognitive trajectories is interesting.

Presence of ADHD in childhood or adolescence was associated with a greater deficit on the sustained attention task but no other cognitive measure, which is in agreement with previous findings^13^. As processing speed and working memory were not associated with presence of ADHD, this lends support to the hypothesis that there may be a specific neurobiological pathway which leads to attention deficits which affect cognition and behaviour^13^. It should be noted that the clinical presentation of ADHD in 22q11.2DS differs from idiopathic ADHD in that it is predominantly the inattentive subtype^48^, so differing relationships with cognition may be seen in other subtypes. In a previous study including a subgroup of participants from the current study (see Supplementary Table 1), there was no relationship between ADHD and cognitive function^2^. Possibly the larger sample size in the current study enabled detection of the association between sustained attention and ADHD due to increased statistical power. The presence of anxiety disorder, however, was not associated with cognitive function in either the current or previous study including some of the same participants^2^, providing further suggestion that these traits may not be linked in 22q11.2DS. Other research which found a relationship between anxiety and working memory impairments^14^ controlled for ADHD as a covariate in the analysis, which may explain different findings.

Adults with 22q11.2DS and psychotic disorder appeared to show greater deficits in attention as has been previously reported^16^. Furthermore, the fact that impairment in attention was present in the sample from a young age and was associated with psychosis supports the hypothesis that attention deficits are a core feature of schizophrenia, both in the high-risk and clinical stages^22^. There were greater deficits in IQ compared to those with no psychotic disorder, which is in line with previous research suggesting that a decline in IQ precedes the emergence of psychotic disorder^6,20^. However, as this study is cross-sectional it cannot be determined if the cognitive impairments precede or result from the onset of psychosis. We did not find deficits in working memory or processing speed which have been previously reported in individuals with 22q11.2DS and psychotic disorder^16^. It could be that in our sample these deficits were general features of 22q11.2DS and not associated with psychosis^21^. Furthermore, in previous studies attention and working memory have been combined into a broader “executive function” domain which was reported as the most striking impairment in individuals with 22q11.2DS and psychosis;^17^ however, as we have shown, there may be differences even within this broad executive function domain, with deficits in attention possibly driving the association with psychotic disorder.

Overall, children with 22q11.2DS displayed a greater deficit in processing speed compared to adults. This supports previous longitudinal research showing that there is developmental maturation in the processing speed domain through adolescence^24^ and it now appears that this maturation is maintained in adulthood. Adults displayed the greatest deficits in working memory which supports previous research which proposed that adults with 22q11.2DS reach a “developmental plateau” in working memory ability before controls, and so may not experience ongoing age-appropriate increases in this domain^28^. That study however did not extend beyond the age of 26, whereas our wide age range up to 60 years old provides insights into the relative strengths and weaknesses in adults with 22q11.2DS more generally, suggesting that some domains such as processing speed may “catch-up”, and others such as working memory may be targets for remediation^49^. Executive performance has been found to be associated with adaptive functioning in adults with 22q11.2DS with or without schizophrenia, lending support to the idea that remediation in executive performance may also benefit functional outcome^18^.

There were greater IQ impairments in adults with 22q11.2DS compared to younger individuals, as has been previously reported^12^ which may be related to presence of psychotic disorder (see above in Discussion section). There were no differences between children and adolescents with 22q11.2DS on IQ measures which contradicts previous work that IQ deficits are larger in adolescents with 22q11.2DS than children^6^. The reason for this discrepancy with previous findings may be a result of including a control group, as previous longitudinal work found that when taking into account control performance, there was no strong evidence for a ‘cognitive decline’ over childhood specific to 22q11.2DS^24^.

### Limitations

The study was multi-site which facilitated a large sample size; furthermore, combining data from different sites may reduce bias from individual sites^50^. However, as the majority of the child and adolescent participants were recruited from Cardiff University, and the majority of adults from the other sites, it could be hypothesised that differences between adults and the child and adolescent group may be attributable to site specific characteristics which influenced cognitive performance. However, adding site as a covariate to the analyses generally did not alter results, except there was a significant difference between children and adolescents on working memory which was previously not significant. Furthermore, it is a strength that we have included adults, as there is far less research on older individuals than paediatric 22q11.2DS samples^31^.

Longitudinal studies have found that rates of psychopathology such as ADHD and psychotic symptoms in 22q11.2DS have variable trajectories and may remit or persist over time^51,52^. The cross-sectional design of the current study was unable to capture this variation in the same individuals over time and therefore associations reported between cognition and psychopathology may differ if measured longitudinally. Furthermore, comorbidity of psychopathology in 22q11.2DS may affect associations between psychopathology and cognition. Comorbidity is a common feature of 22q11.2DS^51,53^. It could be argued that for the analysis of a specific trait, possible overlap at the item level with another trait should be taken into account. This would however, complicate the interpretation of findings at the trait level and also mean that the real-life complex clinical presentation of these young people would no longer be adequately captured. Future studies of children with 22q11.2DS should focus on dimensional measures of psychopathology that capture symptoms that cut across traditional diagnostic categories^1^.

Children with 22q11.2DS were younger on average than child controls, however as the cognitive assessments took age into account we would not expect this to impact interpretation of results. Furthermore, although there were significantly more females in adult individuals with 22q11.2DS than controls running all analyses with gender as a covariate did not change results. As some participants were unable to complete the assessments, it’s possible that our findings may not represent those individuals with the greatest cognitive deficits^8^.

Different versions of cognitive tests were used in older and younger individuals, such as IQ tests, which is a general issue in the field^6^. Therefore it was important for us to include comparison groups, to control for version differences. However, the recruitment of adult sibling controls is fraught with difficulty and therefore our adult control sample consisted of individuals from the community. This may introduce bias as our comparisons for child and adolescence groups consisted of family controls. A meta-analysis reported that comparing the cognitive performance of individuals with 22q11.2DS to community controls produced smaller effect sizes than comparing against control siblings^31^. However, this did not appear to be the case in the current study, with effect sizes of a similar magnitude across developmental stages.

This study made the most of the fact that several studies had used the same measures. In future studies, best practice would be for prospective assessment with the same control recruitment strategy at several sites. Despite this, in rare syndromes such as 22q11.2DS, it is best practice to collaborate and data from different sites has been pooled in many previous studies^20,54^.

### Implications

The findings of the current study support the view that it is essential to consider developmental period (childhood, adolescence, adulthood) when investigating cognitive domains in individuals with 22q11.2DS as observations generated when examining one developmental stage may not apply to others. Furthermore, research linking cognition and psychopathology in 22q11.2DS, and indeed other genetic syndromes, should account for developmental stages in their samples, as grouping children and adolescents together in analyses could mask potential associations. This study also shows that it is important to investigate cognitive ability beyond IQ as presence of ASD and ADHD was not associated with IQ, but was with specific neurocognitive functions, where the underlying mechanisms are more easily interpretable than the general measure of IQ.

An especially important aim for future research is the inclusion of more adults and older individuals with 22q11.2DS where there has been less research than on paediatric populations. It is also important to include comparison or control groups, which could take the form of sibling, IQ-matched peers or typically developing peers, as this enables interpretation of cognitive findings.

There is a need for increased awareness of clinicians of critical periods or windows more amenable to intervention which have been highlighted in this study, such as in children with probable ASD, before greater impairment is present in adolescence. Encouragingly, cognitive remediation has been found to be feasible and effective in adolescents with 22q11.2DS^49^, and may be especially successful at these periods.

## Supplementary information

Supplementary Tables and Figures
