# Supplementary material for: Cognitive deficits in childhood, adolescence and adulthood in 22q11.2 deletion syndrome and association with psychopathology
Source: Transl Psychiatry. 2020 Feb 3;10:53. doi: 10.1038/s41398-020-0736-7 (PMC7026075; doi:10.1038/s41398-020-0736-7)
Supplement: Supplementary file 1 — Supplementary Tables and Figures [file 41398_2020_736_MOESM1_ESM.docx]

Supplementary Table 1. Site specific sample sizes, ascertainment methods and exclusion criteria.

|  |  |  | *Site* | | | | | | | | |
| --- | --- | --- | --- | --- | --- | --- | --- | --- | --- | --- | --- |
|  |  | *Cardiff* | | |  | *Leuven* |  | *Maastricht* | | | *Total* |
|  |  | n | *Ascertainment* | *Exclusion* | *n* | *Ascertainment* | *Exclusion* | *n* | *Ascertainment* | *Exclusion* | *n* |
| *Children (6-10 years)* | 22q11.2DS | 60* | Medical genetics clinics, charities, social media | None | - | - | - | - | - | - | 60 |
|  | Control siblings | 23* | Sibling of individual with 22q11.2DS | No diagnosis of 22q11.2DS | - | - | - | - | - | - | 23 |
| *Adolescents (10-18 years)* | 22q11.2DS | 61* | Medical genetics clinics, charities, social media | None | 5 | Medical genetics service | None | - | - | - | 66 |
|  | Control siblings | 35* | Sibling of individual with 22q11.2DS | No diagnosis of 22q11.2DS | - | - | - | - | - | - | 35 |
| *Adults (18+ years)* | 22q11.2DS | 18 | Medical genetics clinics, charities, social media | None | 28 | Medical genetics service | None | 64 | Family associations, academic psychiatry outpatient clinic (automatically referred after diagnosis of 22q11.2DS, independently from any psychiatric problems) | None | 110 |
| *(18+ years)* | Community controls | - | - | - | - | - | - | 48 | Advertisement on internet | 18-60 years of age, not using psychotropic medication, no history of psychiatric illness, no first degree relative with 22q11.2DS | 48 |

* 80/121 (66%) of these children and adolescents with 22q11.2DS and 39/58 (67%) siblings were included in the Niarchou, Zammit [1] study. Similarities and differences between results are discussed in the Discussion.

*Supplementary Table 2. Age and gender distributions in 22q11.2DS and controls across developmental stages*

|  |  | *n* | *Age; mean (sd)* | *t (df)* | *p* | *Effect size; Cohen’s d* | *Females; n (%)* | *X^2^ (df)* | *p* | *Effect size; Cramer's V* |
| --- | --- | --- | --- | --- | --- | --- | --- | --- | --- | --- |
| *Children (6-10 years)* | 22q11.2DS | 60 | 8.15 (0.90) | 2.32 (81) | **0.023*** | 0.57 | 29 (48%) | 0 | 1 | 0 |
|  |  |  |  |  |  |  |  |  |  |  |
|  | Control siblings | 23 | 8.73 (1.05) |  |  |  | 11 (48%) |  |  |  |
|  |  |  |  |  |  |  |  |  |  |  |
| *Adolescents (10-18 years)* | 22q11.2DS | 66 | 12.5 (1.93) | 1.12 (99) | 0.267 | 0.23 | 33 (50%) | 0.04 (1) | 0.841 | 0.02 |
|  | Control siblings | 35 | 12.08 (1.61) |  |  |  | 16 (46%) |  |  |  |
| *Adults (18+ years)* | 22q11.2DS | 110 | 30.49 (10.33) | 1.98 (126.84) | 0.05 | 0.30 | 74 (67%) | 5.57 (1) | **0.018*** | 0.19 |
|  |  |  |  |  |  |  |  |  |  |  |
|  | Community controls | 48 | 27.67 (7.12) |  |  |  | 22 (46%) |  |  |  |
|  |  |  |  |  |  |  |  |  |  |  |

*Supplementary Table 3. Medication use.*

|  | Neuroactive and thyroid medication | n (adults) |
| --- | --- | --- |
|  | Quetiapin | 2 |
|  | Quetiapin and Aripiprazole | 1 |
|  | Aripiprazole | 2* |
|  | Risperidone | 3 |
|  | Alprazolam and Sulpiride | 1 |
|  | Priadel, Strattera and Zyprexa | 1 |
|  | Abilify, Depakine and Levothyroxine | 1 |
|  | Aripiprazole | 1 |
|  | Seroquel | 1 |
|  | Lorazepam, Abilify, Seroquel and Clozapine | 1 |
|  | Clozapine | 2 |
|  | Haloperidol | 1 |
|  | Fluoxetine and Antipsychotic Not specified | 1 |
|  | Mirtazapine and Olanzapine | 1 |
|  | Sertraline | 2 |
|  | Paroxetine, Zolpidem and Risperidone | 1 |
|  | Levetiracetam | 1 |
|  | Escitalopram and Bupopion | 1 |
|  | Escitalopram, Prothiphendyl and Bromazepam | 1 |
|  | Escitalopram | 1 |
|  | Paroxetine | 4 |
|  | Methylphenidate | 2 |
|  | Oxazepam | 1 |
|  | Alprazolam and Venlafaxine | 1 |
|  | Unknown antiepileptic | 1 |
|  | Citalopram and Levothyroxine | 1 |
|  | Citalopram and Seroquel | 1 |
|  | Diazepam, Amytriptyline, Paroxetine | 1 |
|  | Clomipramine and Seroquel | 1 |
|  | Propranolol | 1 |
|  | Fluoxetine, Topiramate, Acetazolamide, Aripiprazole and Levothyroxine | 1 |
|  | Fluoxetine and Gabapentin | 1 |
|  | Citalopram and Risperidone | 1 |
|  | Gabapentin | 1 |
|  | Epilem | 1* |
|  | Sodium valproate | 1* |
|  | Levothyroxine | 7† |

* n includes 1 adolescent

† n includes 1 child

*Supplementary Table 4. Cognitive functions assessed by CANTAB tests.*

| Neurocognitive function | Test name | Task outline | Raw measure |
| --- | --- | --- | --- |
| Processing speed | Five Choice Reaction Time (RTI) | Participants must respond as fast as possible to a stimulus in one of five locations | Reaction time (ms) |
| Sustained attention | Rapid Visual Information Processing (RVP) | Participants must respond to a target sequence of digits over a few minutes of a continuous pseudo-random presentation of digits | A’ (A prime); probability of correct responses |
| Spatial working memory | Spatial Working Memory (SWM) | Participants must remember where previous targets were in space | Between errors; the number of times the participant returned to a box where a target had already previously been found |

*Supplementary Table 5. Cognitive and psychiatric assessments across sites. Psychiatric instruments and*

|  |  |  |  |  |  |  |  |  |
| --- | --- | --- | --- | --- | --- | --- | --- | --- |
|  | *Cardiff* | | | *Leuven* | *Maastricht* | | | |
|  | *Psychiatric* | | *Cognitive* | *Psychiatric* | | *Cognitive* | *Psychiatric* | *Cognitive* |
| Children and adolescents (6-18 years) | CAPA (psychotic experiences/disorders, ADHD and anxiety disorder) [2], Child CAPA (child self-report of psychotic experiences/disorders and anxiety disorder) [2], SCQ (probable autism spectrum disorder) [3] | | CANTAB [4], WASI [5] | N/A | | CANTAB [4], WISC-III [6] | - | - |
| Adults  (18+ years) | SIPS (prodromal psychotic symptoms) [7], PAS-ADD (psychotic disorder) [8] | | CANTAB [4], WASI [5] | CAARMS (prodromal psychotic symptoms) [9], SCID [10] and MINI plus (psychotic disorder) [11] | | CANTAB [4], WAIS-III [12] | PQ-16 (prodromal psychotic symptoms) [13], MINI (psychotic disorder) [11] | CANTAB [4], WAIS-III [12] |

1. Niarchou, M., et al., *Psychopathology and cognition in children with 22q11.2 deletion syndrome.* Br J Psychiatry, 2014. **204**(1): p. 46-54.

2. Angold, A., et al., *The Child and Adolescent Psychiatric Assessment (CAPA).* Psychol Med, 1995. **25**(4): p. 739-53.

3. Rutter, M.B.L., C., *Social Communication Questionnaire*. 2003: Western Psychological Services.

4. CANTAB, *CANTAB Eclipse Version 3*. 2006, Cambridge Cognition.

5. Wechsler, D., *Wechsler Abbreviated Scale of Intelligence–Second Edition (WASI-II)*. 2011, San Antonio, TX: NCS Pearson.

6. Wechsler, D., *Wechsler Intelligence Scale for Children*. 1991, San Antonio, TX: The Psychological Corporation.

7. McGlashan, T.H., et al., *Instrument for the Assessment of Prodromal Symptoms and States*, in *Early Intervention in Psychotic Disorders*, T. Miller, et al., Editors. 2001, Springer Netherlands: Dordrecht. p. 135-149.

8. Moss, S., et al., *Psychiatric morbidity in older people with moderate and severe learning disability. I: Development and reliability of the patient interview (PAS-ADD).* Br J Psychiatry, 1993. **163**: p. 471-80.

9. Yung, A.R., et al., *Mapping the onset of psychosis: the Comprehensive Assessment of At-Risk Mental States.* Aust N Z J Psychiatry, 2005. **39**(11-12): p. 964-71.

10. First, M.B., Spitzer, R. L., Gibbon, M., & Williams, J. B. W., *Structured clinical interview for DSM-IV-TR axis I disorders, research version, patient edition. (SCID-I/P).* 2002: New York: Biometrics Research, New York State Psychiatric Institute.

11. Sheehan, D.V., et al., *The Mini-International Neuropsychiatric Interview (M.I.N.I.): the development and validation of a structured diagnostic psychiatric interview for DSM-IV and ICD-10.* J Clin Psychiatry, 1998. **59 Suppl 20**: p. 22-33;quiz 34-57.

12. Wechsler, D., *Wechsler Adult Intelligence Scale—third edition*. 1997, San Antonio, TX: The Psychological Corporation.

13. Ising, H.K., et al., *The validity of the 16-item version of the Prodromal Questionnaire (PQ-16) to screen for ultra high risk of developing psychosis in the general help-seeking population.* Schizophrenia bulletin, 2012. **38**(6): p. 1288-1296.

Supplementary Table 6. Prevalence of probable ASD, ADHD and anxiety disorder in children and adolescents with 22q11.2DS and typically developing control siblings.

|  | *Child* | | | | | *Adolescent* | | | | |
| --- | --- | --- | --- | --- | --- | --- | --- | --- | --- | --- |
|  | *22q11.2DS* | *Control* | *X^2^ (df)* | *p* | *Effect size; φc* | *22q11.2DS* | *Control* | *X^2^ (df)* | *p* | *Effect size; φc* |
|  | *n/total (%)* | *n/total (%)* |  |  |  | *n/total (%)* | *n/total (%)* |  |  |  |
| *Probable ASD diagnosis* | 17/58 (29%) | 1/22 (5%) | 4.28 (1) | **0.039*** | 0.23 | 24/63 (38%) | 1/34 (3%) | 12.49 (1) | **<0.001*** | 0.36 |
| *ADHD diagnosis* | 28/59 (47%) | 1/19 (5%) | 9.22 (1) | **0.002*** | 0.34 | 20/63 (32%) | 1/35 (3%) | 9.50 (1) | **0.002*** | 0.31 |
| *Anxiety disorder diagnosis* | 19/60 (32%) | 0/19 (0%) | 6.28 (1) | **0.012*** | 0.28 | 15/64 (23%) | 2/35 (6%) | 3.83 (1) | 0.05 | 0.2 |

Supplementary Table 7. Cognitive performance in children and adolescents with 22q11.2DS relative to typically developing controls, with and without probable ASD.

|  | **Child** | | | | **Adolescent** | | | |
| --- | --- | --- | --- | --- | --- | --- | --- | --- |
|  | ***Probable ASD*** | | ***No ASD*** | | ***Probable ASD*** | | ***No ASD*** | |
|  | **n** | **Mean (sd)** | **n** | **Mean (sd)** | **n** | **Mean (sd)** | **n** | **Mean (sd)** |
| **Neurocognitive scores** |  |  |  |  |  |  |  |  |
| Processing speed | 14 | -0.80 (3.09) | 39 | -1.41 (1.84) | 24 | -1.16 (2.08) | 34 | -0.03 (0.85) |
| Sustained attention | 13 | -1.53 (2.06) | 38 | -2.53 (2.62) | 18 | -3.66 (7.01) | 32 | -0.92 (1.19) |
| Working memory | 17 | -0.81 (1.05) | 41 | -1.25 (1.00) | 24 | -1.07 (0.84) | 38 | -0.49 (0.80) |
| **IQ test scores** |  |  |  |  |  |  |  |  |
| Full-scale IQ | 17 | -34.97 (9.74) | 39 | -34.88 (10.49) | 24 | -33.86 (12.72) | 38 | -30.33 (13.69) |
| Verbal IQ | 17 | -36.75 (12.13) | 39 | -33.87 (12.52) | 24 | -32.63 (11.87) | 38 | -27.93 (14.58) |
| Performance IQ | 17 | -26.26 (10.25) | 41 | -29.24 (8.98) | 24 | -30.21 (13.45) | 38 | -28.09 (13.05) |

*Within each developmental stage (children and adolescents) the mean score for control siblings was subtracted from the score of each individual with 22q11.2DS for each cognitive measure, producing the following cognitive difference scores for children and adolescents with 22q11.2DS with or without probable ASD. A difference of 0 would therefore represent no difference between the individual with 22q11.2DS and the control mean; a negative difference represents an impairment on that measure in the individual with 22q11.2DS compared to the mean control performance.*

Supplementary Table 8. Cognitive performance in children and adolescents with 22q11.2DS relative to typically developing controls, with and without ADHD.

|  | **Child** | | | | **Adolescent** | | | |
| --- | --- | --- | --- | --- | --- | --- | --- | --- |
|  | ***ADHD*** | | ***No ADHD*** | | ***ADHD*** | | ***No ADHD*** | |
|  | **n** | **Mean (sd)** | **n** | **Mean (sd)** | **n** | **Mean (sd)** | **n** | **Mean (sd)** |
| **Neurocognitive scores** |  |  |  |  |  |  |  |  |
| Processing speed | 25 | -1.17 (2.61) | 29 | -1.22 (1.84) | 17 | -1.26 (2.27) | 40 | -0.24 (1.30) |
| Sustained attention | 24 | -3.07 (2.40) | 25 | -1.60 (2.56) | 16 | -4.16 (7.29) | 33 | -0.92 (1.21) |
| Working memory | 28 | -1.22 (1.12) | 31 | -0.98 (0.94) | 19 | -0.84 (1.04) | 43 | -0.66 (0.77) |
| **IQ test scores** |  |  |  |  |  |  |  |  |
| Full-scale IQ | 28 | -32.39 (10.38) | 29 | -36.78 (9.40) | 20 | -33.44 (12.34) | 42 | -30.32 (14.03) |
| Verbal IQ | 28 | -32.37 (13.13) | 29 | -36.42 (11.37) | 20 | -31.54 (13.03) | 42 | -27.82 (14.06) |
| Performance IQ | 28 | -25.98 (8.92) | 31 | -30.08 (9.44) | 20 | -30.47 (12.14) | 42 | -28.17 (13.93) |

*Within each developmental stage (children and adolescents) the mean score for control siblings was subtracted from the score of each individual with 22q11.2DS for each cognitive measure, producing the following cognitive difference scores for children and adolescents with 22q11.2DS with or without ADHD. A difference of 0 would therefore represent no difference between the individual with 22q11.2DS and the control mean; a negative difference represents an impairment on that measure in the individual with 22q11.2DS compared to the mean control performance.*

Supplementary Table 9. Cognitive performance in children and adolescents with 22q11.2DS relative to typically developing controls, with and without anxiety disorder.

|  | **Child** | | | | **Adolescent** | | | |
| --- | --- | --- | --- | --- | --- | --- | --- | --- |
|  | ***Anxiety*** | | ***No Anxiety*** | | ***Anxiety*** | | ***No Anxiety*** | |
|  | **n** | **Mean (sd)** | **n** | **Mean (sd)** | **n** | **Mean (sd)** | **n** | **Mean (sd)** |
| **Neurocognitive scores** |  |  |  |  |  |  |  |  |
| Processing speed | 17 | -0.96 (2.83) | 38 | -1.34 (1.88) | 13 | -0.70 (1.85) | 45 | -0.51 (1.65) |
| Sustained attention | 14 | -2.21 (2.30) | 35 | -2.28 (2.42) | 13 | -1.89 (2.03) | 42 | -1.67 (4.78) |
| Working memory | 19 | -1.16 (1.14) | 41 | -1.07 (0.97) | 14 | -1.04 (0.99) | 49 | -0.62 (0.79) |
| **IQ test scores** |  |  |  |  |  |  |  |  |
| Full-scale IQ | 19 | -35.55 (6.57) | 39 | -34.42 (11.42) | 14 | -32.84 (15.17) | 49 | -31.12 (13.07) |
| Verbal IQ | 19 | -36.31 (9.83) | 39 | -33.84 (13.43) | 14 | -31.30 (12.39) | 49 | -28.64 (14.15) |
| Performance IQ | 19 | -27.41 (7.46) | 41 | -28.58 (10.08) | 14 | -29.74 (15.86) | 49 | -28.84 (12.59) |

*Within each developmental stage (children and adolescents) the mean score for control siblings was subtracted from the score of each individual with 22q11.2DS for each cognitive measure, producing the following cognitive difference scores for children and adolescents with 22q11.2DS with or without anxiety disorder. A difference of 0 would therefore represent no difference between the individual with 22q11.2DS and the control mean; a negative difference represents an impairment on that measure in the individual with 22q11.2DS compared to the mean control performance.*

Supplementary Table 10. Cognitive performance in adults with 22q11.2DS relative to typically developing controls, with and without psychotic disorder.

|  | **Psychotic disorder** | | **No Psychotic disorder** | |
| --- | --- | --- | --- | --- |
|  | **n** | **Mean (sd)** | **n** | **Mean (sd)** |
| **Neurocognitive scores** |  |  |  |  |
| Processing speed | 15 | -0.74 (1.43) | 84 | -0.49 (1.45) |
| Sustained attention | 12 | -3.36 (1.42) | 74 | -2.03 (0.96) |
| Working memory | 15 | -1.56 (0.91) | 91 | -1.62 (0.89) |
| **IQ test scores** |  |  |  |  |
| Full-scale IQ | 16 | -44.48 (10.04) | 88 | -36.76 (11.97) |
| Verbal IQ | 15 | -44.04 (12.52) | 87 | -33.60 (14.36) |
| Performance IQ | 15 | -45.28 (15.69) | 87 | -36.53 (15.40) |

*The mean score for community controls was subtracted from the score of each individual with 22q11.2DS for each cognitive measure, producing the following cognitive difference scores for adults with 22q11.2DS with or without psychotic disorder. A difference of 0 would therefore represent no difference between the individual with 22q11.2DS and the control mean; a negative difference represents an impairment on that measure in the individual with 22q11.2DS compared to the mean control performance.*

Supplementary Table 11. Cognitive performance across developmental stages in individuals with 22q11.2DS relative to typically developing controls.

|  | **Child** | | **Adolescent** | | **Adult** | |  |
| --- | --- | --- | --- | --- | --- | --- | --- |
|  | **N** | **Mean (sd)** | **n** | **Mean (sd)** | **n** | **Mean (sd)** | |
| **Neurocognitive scores** |  |  |  |  |  |  | |
| Processing speed | 55 | -1.22 (2.19) | 60 | -0.53 (1.66) | 99 | -0.53 (1.44) | |
| Sustained attention | 49 | -2.26 (3.65) | 56 | -1.68 (4.24) | 86 | -2.21 (1.12) | |
| Working memory | 60 | -1.10 (1.02) | 65 | -0.71 (0.84) | 106 | -1.61 (0.89) | |
| **IQ test scores** |  |  |  |  |  |  | |
| Full-scale IQ | 58 | -34.79 (10.05) | 65 | -31.59 (13.35) | 104 | -37.95 (11.98) | |
| Verbal IQ | 58 | -34.65 (12.33) | 65 | -29.38 (13.80) | 102 | -35.13 (14.53) | |
| Performance IQ | 60 | -28.21 (9.29) | 65 | -29.05 (13.06) | 102 | -37.82 (15.68) | |

Within each developmental stage (children, adolescents and adults) the mean score for the control sample (typically developing siblings for the children and adolescents and community controls for the adults) was subtracted from the score of each individual with 22q11.2DS for each cognitive measure. This produced a difference score for each individual with 22q11.2DS on each measure. Mean cognitive difference scores on neurocognitive and IQ measures for each developmental stage with sd are displayed. A difference of 0 would represent no difference between the individual with 22q11.2DS and the control mean. A negative difference represents an impairment on that measure in individuals 22q11.2DS compared to the mean control performance.

Supplementary Figure 1. Association of probable ASD with neurocognitive performance in children and adolescents with 22q11.2DS relative to typically developing controls.


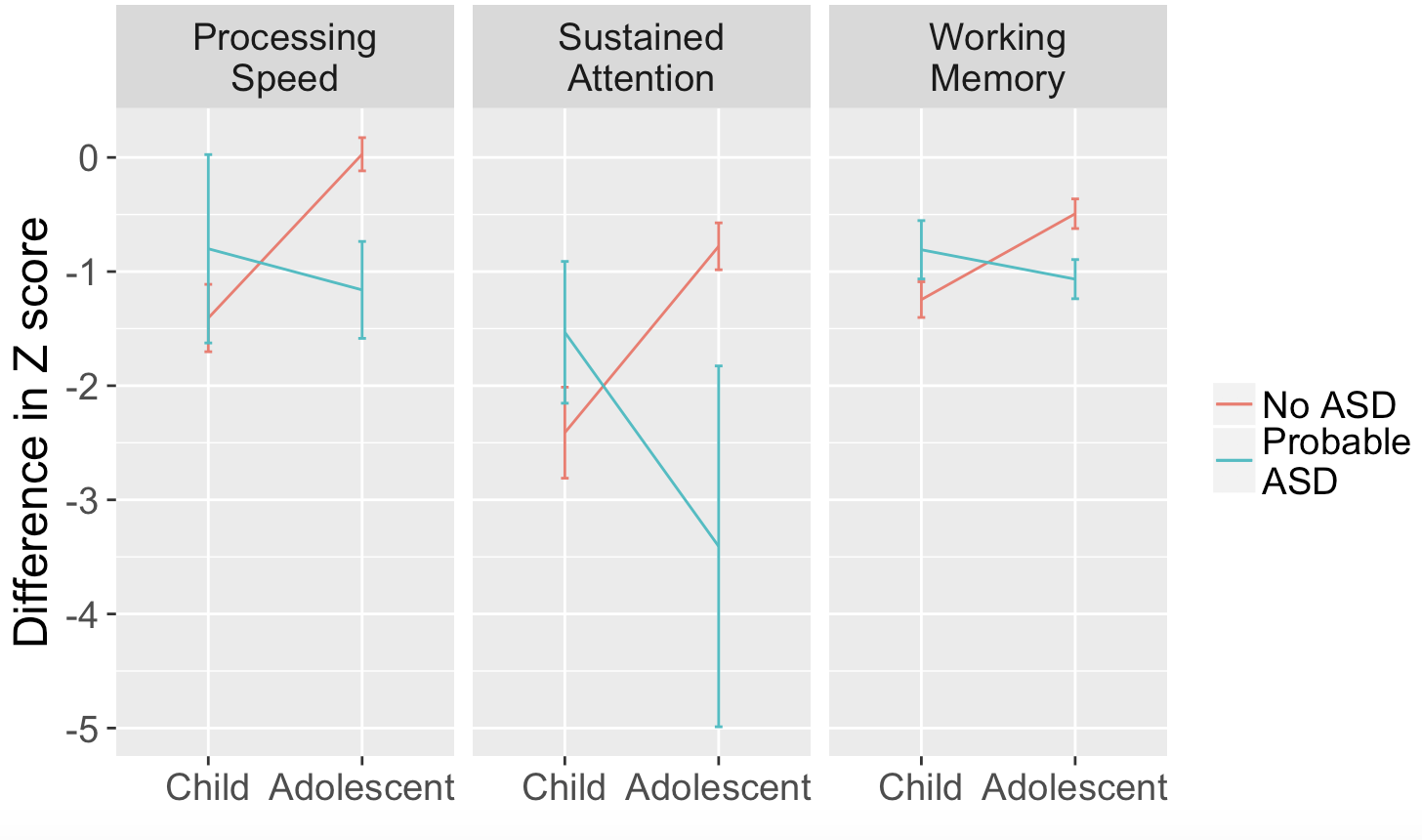


*

*

*

p=0.021

p=0.022

p=0.006

*ASD, Autism Spectrum Disorder.*

Supplementary Figure 2. Association of ADHD with sustained attention in children and adolescents with 22q11.2DS relative to typically developing controls.


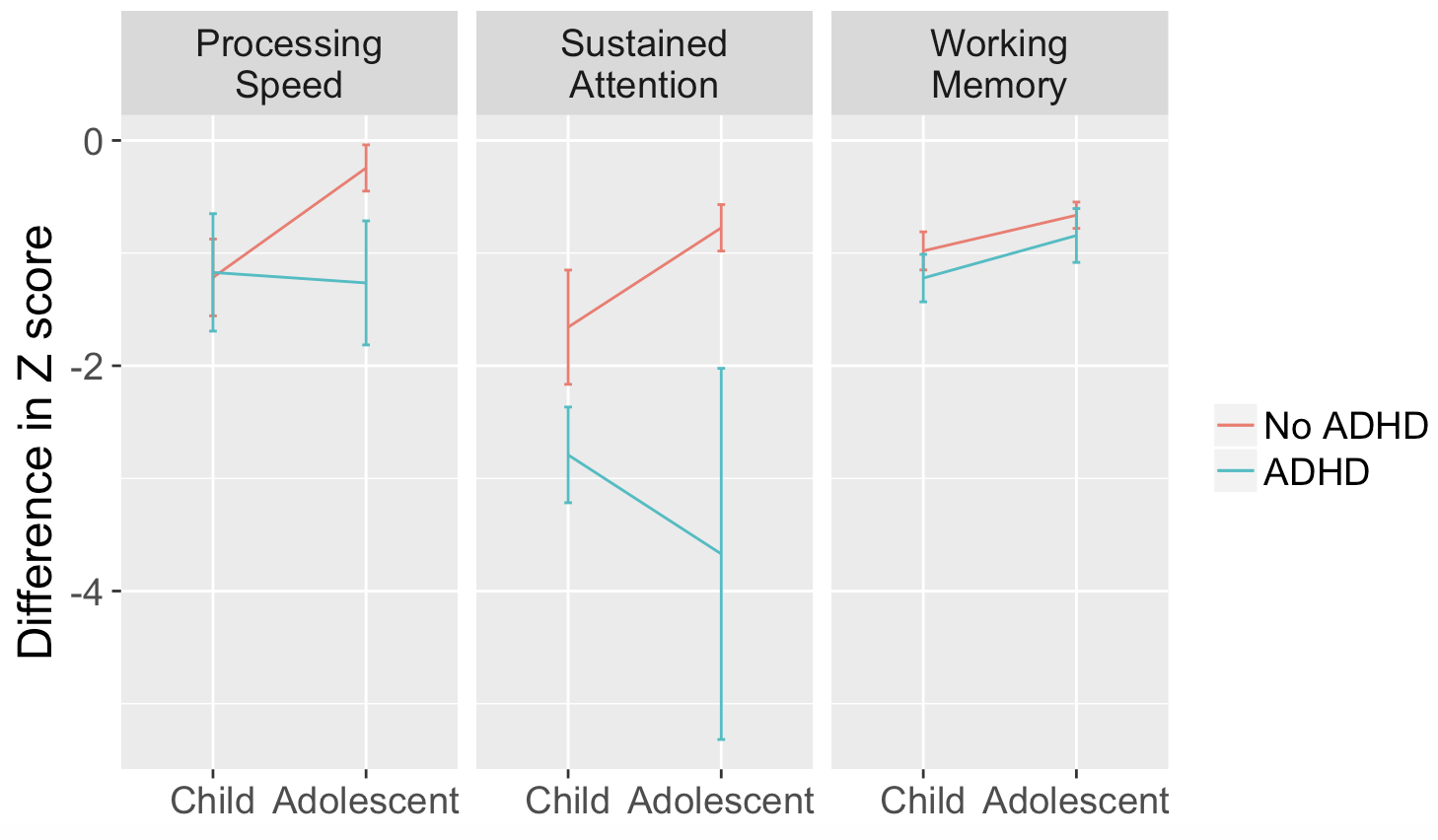

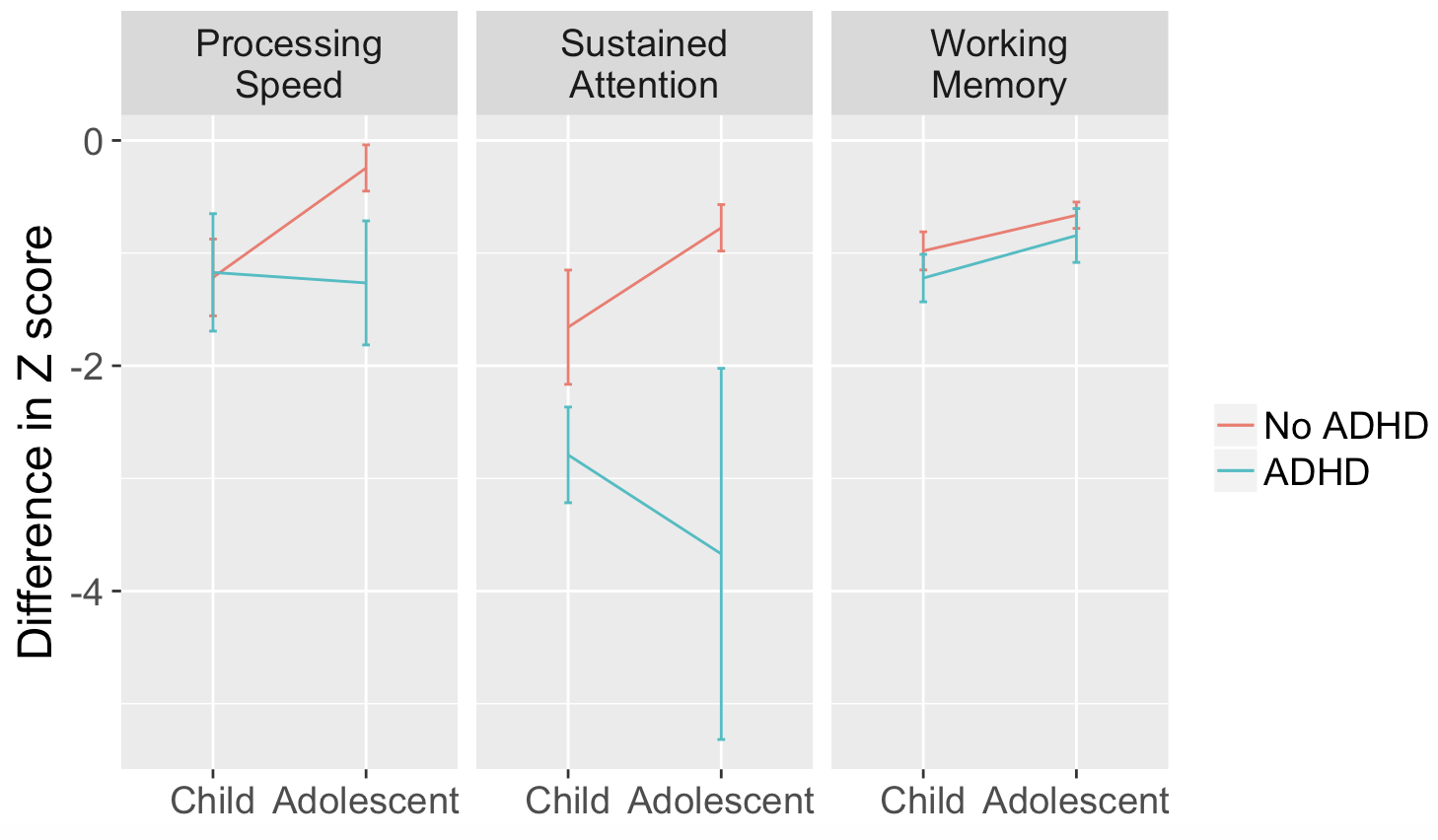

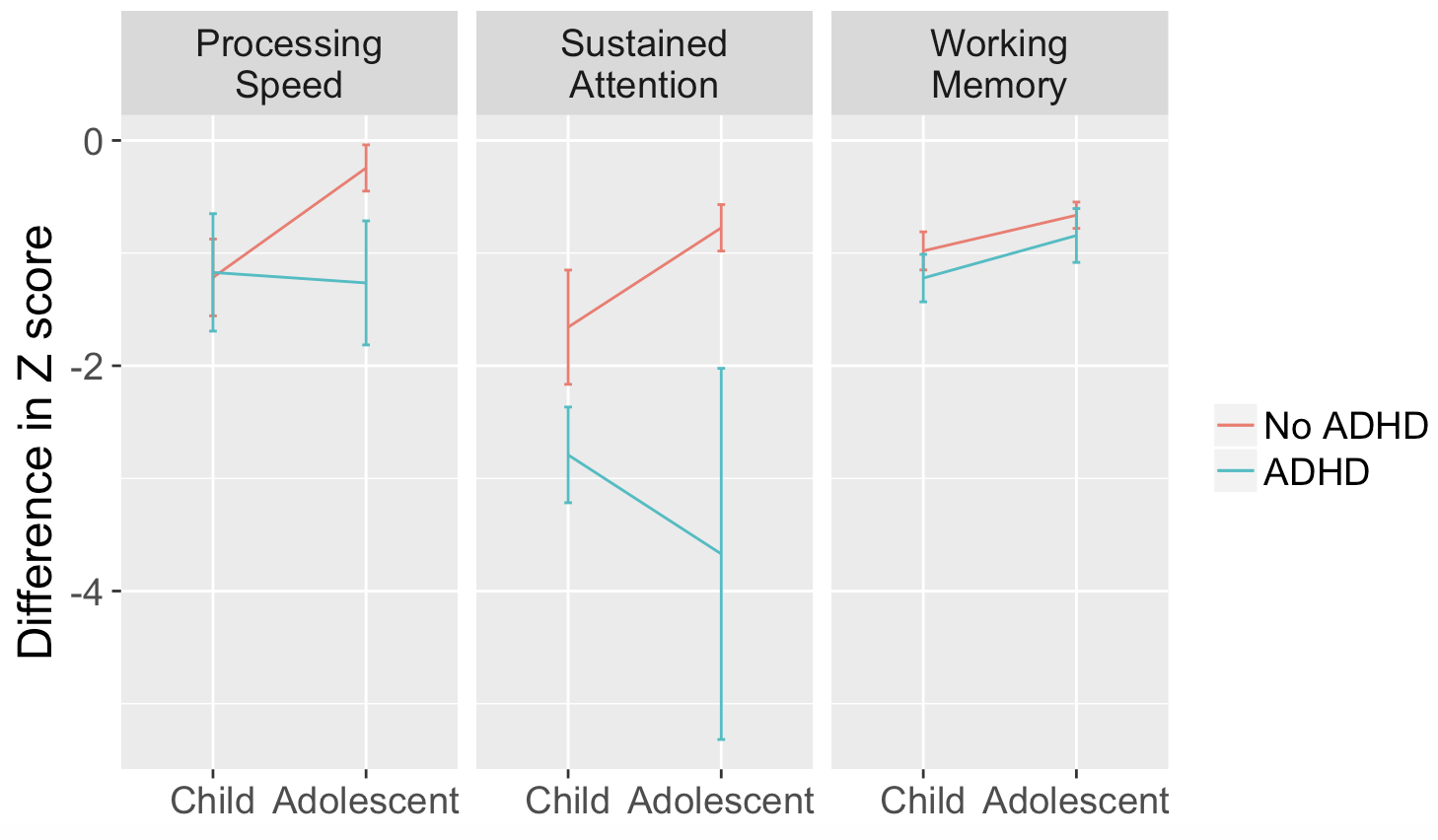


(p=0.004)

*

ADHD, Attention Deficit Hyperactivity Disorder.

Supplementary Figure 3. Association of psychotic disorder with neurocognitive performance in adults with 22q11.2DS relative to typically developing controls.


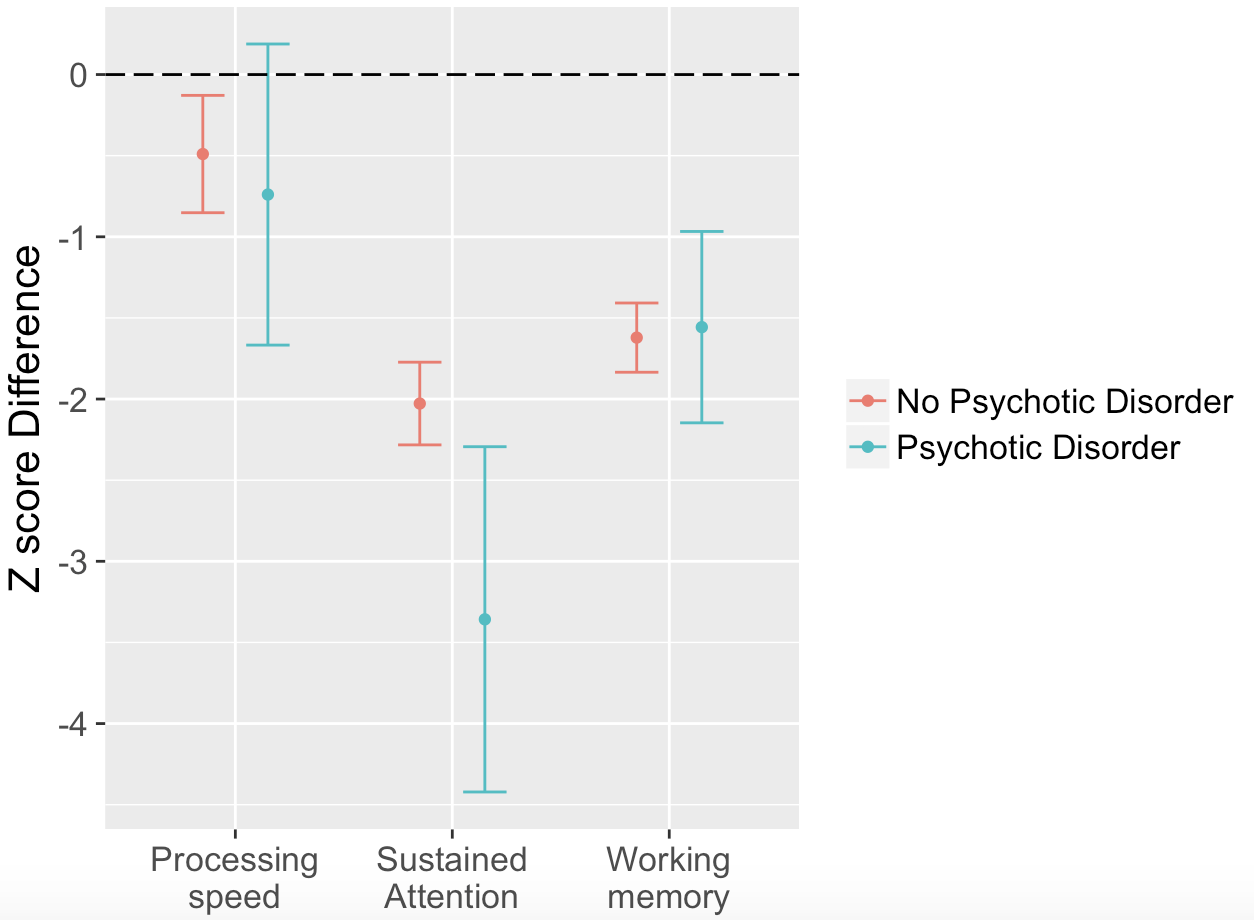


*

Supplementary Figure 4. Association of psychotic disorder with IQ in adults with 22q11.2DS relative to typically developing controls.


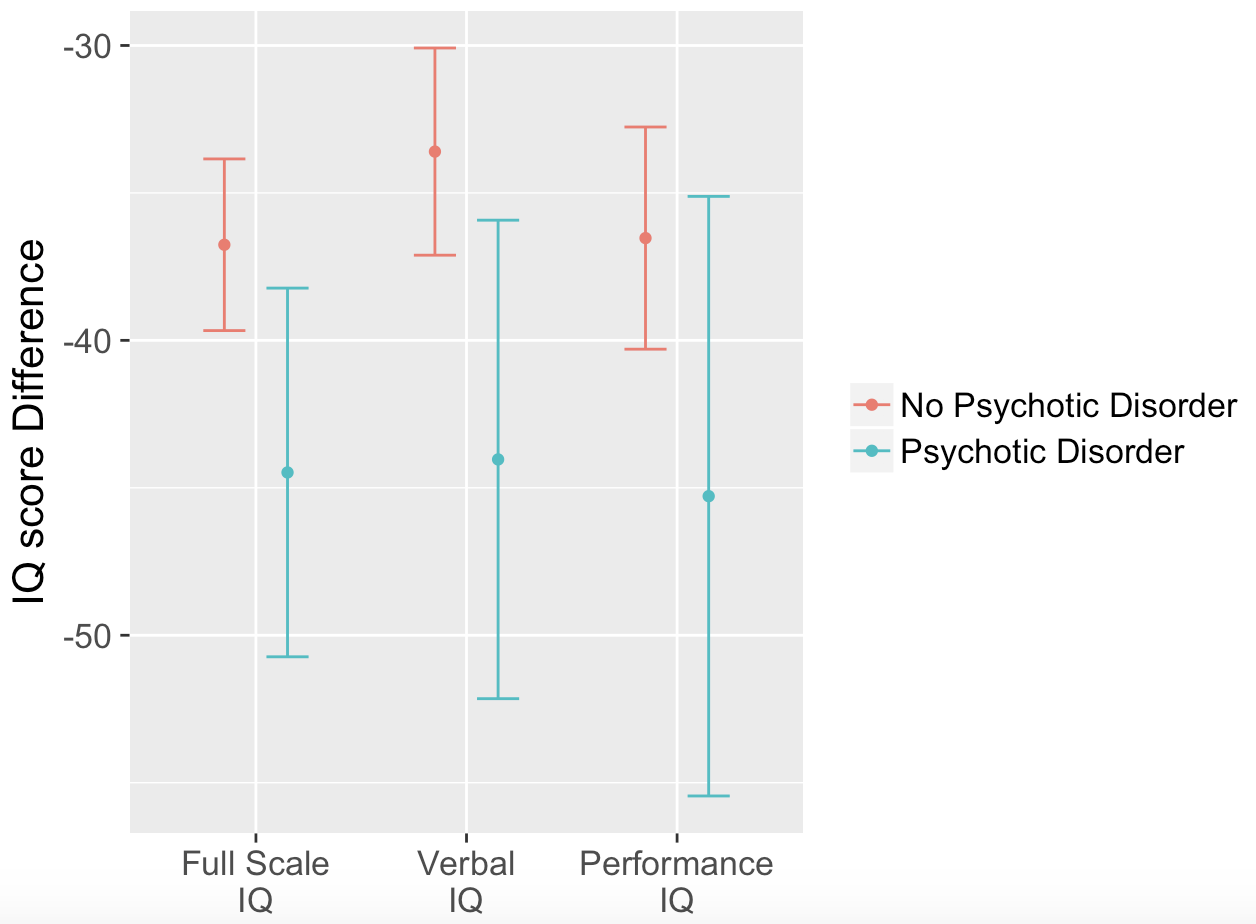


*

*

*

Supplementary Figure 5. Neurocognitive performance across developmental stages in individuals with 22q11.2DS relative to typically developing controls.


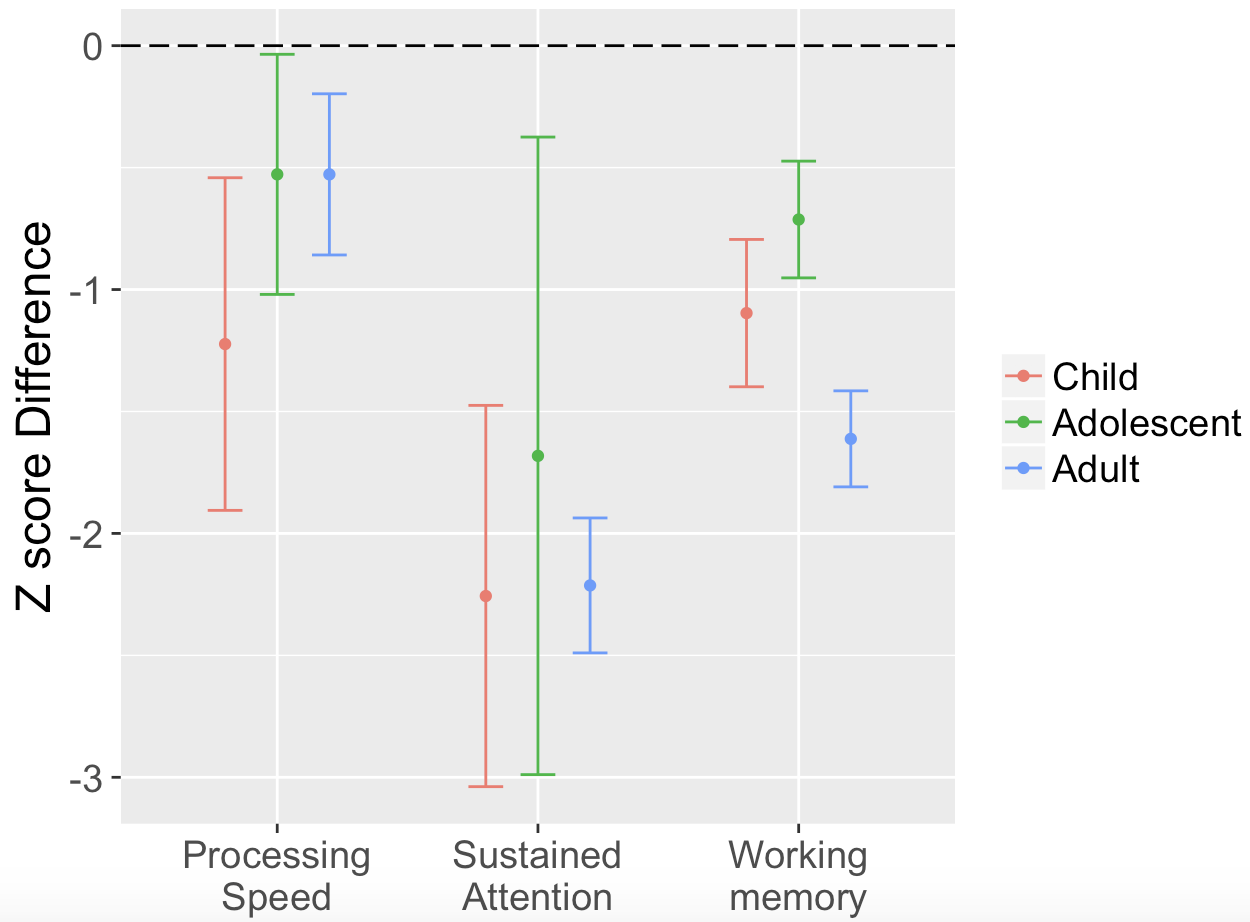


*

*

p=0.002

p<0.001

*

p=0.045

Supplementary Figure 6. IQ performance across developmental stages in individuals with 22q11.2DS relative to typically developing controls.


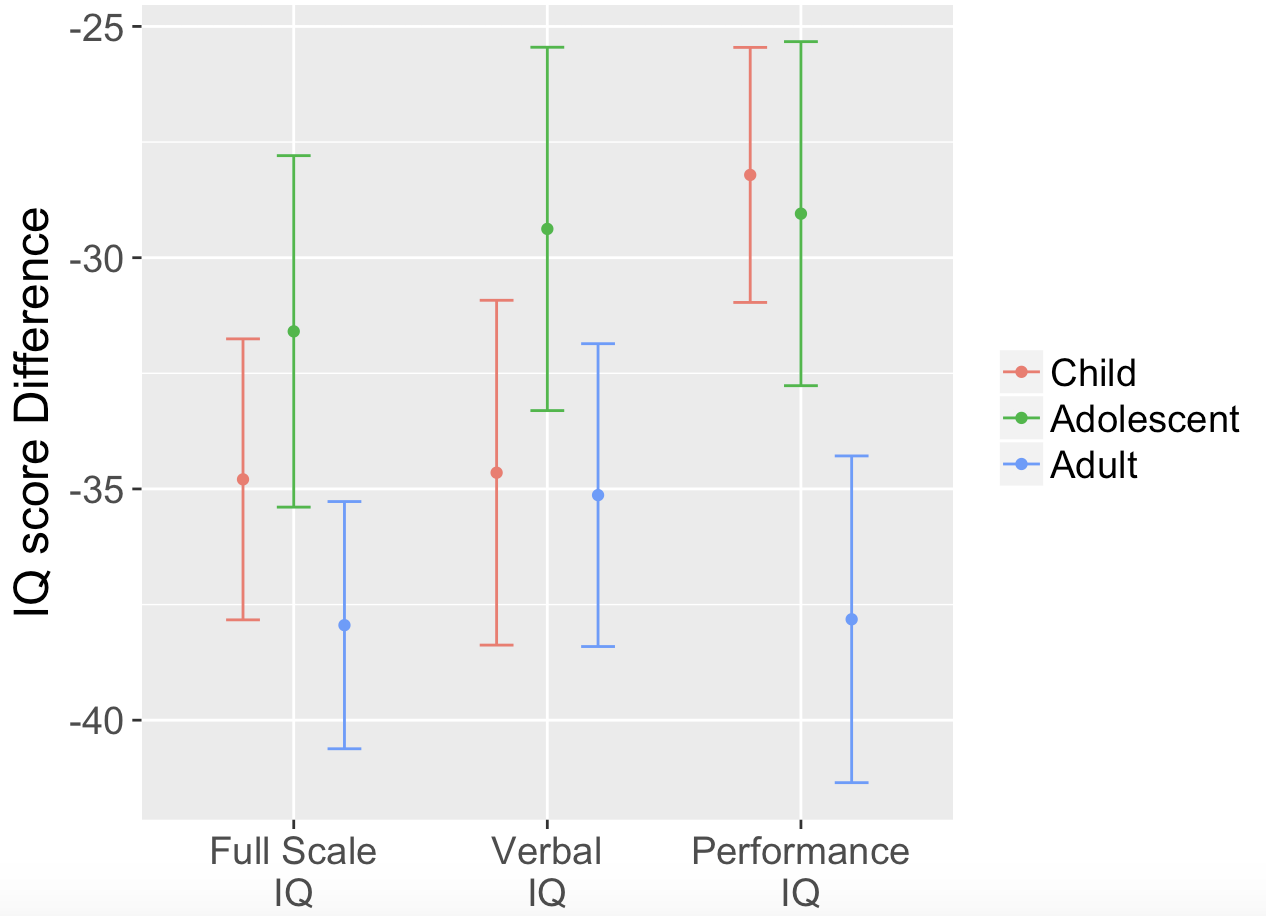


*

p<0.001

*

p<0.001

*

p=0.025

*

p=0.003
